# Design and management of public health outreach using interoperable mobile multimedia: an analysis of a national winter weather preparedness campaign

**DOI:** 10.1186/s12889-016-3104-z

**Published:** 2016-05-25

**Authors:** Cesar Bandera

**Affiliations:** Martin Tuchman School of Management, New Jersey Institute of Technology, 4031 Central Avenue Building, University Heights, Newark, New Jersey 07102 USA

**Keywords:** Text messaging, Mobile multimedia, Emergency preparedness, m-health, Outreach

## Abstract

**Background:**

The Office of Public Health Preparedness and Response (OPHPR) in the Centers for Disease Control and Prevention conducts outreach for public preparedness for natural and manmade incidents. In 2011, OPHPR conducted a nationwide mobile public health (m-Health) campaign that pushed brief videos on preparing for severe winter weather onto cell phones, with the objective of evaluating the interoperability of multimedia m-Health outreach with diverse cell phones (including handsets without Internet capability), carriers, and user preferences.

**Methods:**

Existing OPHPR outreach material on winter weather preparedness was converted into mobile-ready multimedia using mobile marketing best practices to improve audiovisual quality and relevance. Middleware complying with opt-in requirements was developed to push nine bi-weekly multimedia broadcasts onto subscribers’ cell phones, and OPHPR promoted the campaign on its web site and to subscribers on its govdelivery.com notification platform. Multimedia, text, and voice messaging activity to/from the middleware was logged and analyzed.

**Results:**

Adapting existing media into mobile video was straightforward using open source and commercial software, including web pages, PDF documents, and public service announcements. The middleware successfully delivered all outreach videos to all participants (a total of 504 videos) regardless of the participant’s device. 54 % of videos were viewed on cell phones, 32 % on computers, and 14 % were retrieved by search engine web crawlers. 21 % of participating cell phones did not have Internet access, yet still received and displayed all videos. The time from media push to media viewing on cell phones was half that of push to viewing on computers.

**Conclusions:**

Video delivered through multimedia messaging can be as interoperable as text messages, while providing much richer information. This may be the only multimedia mechanism available to outreach campaigns targeting vulnerable populations impacted by the digital divide. Anti-spam laws preserve the integrity of mobile messaging, but complicate campaign promotion. Person-to-person messages may boost enrollment.

## Background

Exploiting the ubiquitous adoption of cell phones, government and commercial entities are delivering services via cell phones, including via text messaging [1, 2]. Healthcare is likewise increasingly delivered via cell phones, resulting in the growing field of m-Health [3, 4] and an increased use of text messaging in public health outreach campaigns due to its low cost, ubiquity, and reliability [5, 6]. In contrast, high cost and playback unreliability have impeded the use of mobile video in a similar capacity in spite of the well-known educational and motivational benefits of multimedia over simple text [7–9]. These impediments have also prevented demographics without Internet access from accessing the abundance of web-based multimedia on health topics [10].

The Centers for Disease Control and Prevention (CDC) maintain a comprehensive web site with public health information, and the CDC Office of Public Health Preparedness and Response (OPHPR) is responsible for the subset that covers preparedness for natural and manmade incidents. Because these incidents disproportionally impact poor demographics [11], OPHPR is investigating m-Health outreach mechanisms that can push multimedia to demographics that lack Internet access or computer literacy [12].

### Research question

This paper empirically addresses the question: can m-Health outreach campaigns use multimedia as an alternative to text messaging without sacrificing reach or ease of use. In this study, “reach” means that public preparedness multimedia is received by and playable on all the cell phones of the target audience. Whereas certain apps installed on certain phones can receive and play videos, the study imposes no restrictions on the model of the phone or its configuration (i.e., not requiring any specific carrier, operating system, or any apps to be installed), nor on the participant’s plan (i.e.., pre-paid, subscription, or issued, with or without Internet access). “Ease of use” means the ease with which participants can enroll in the outreach campaign and view videos, and the ease with which subject matter experts develop mobile-ready videos from existing multimedia outreach assets, particularly assets from web-based outreach. The remainder of the Background section describes the motivation behind dropping restrictions on phone types, and the resulting technological challenges impeding the broad reach of mobile multimedia.

### Underutilization of mobile multimedia messaging by m-Health

The ubiquity, resilience, and popularity of mobile text messaging (i.e., Short Message Service, or SMS) have prompted its use in public health preparedness and emergency response. DHHS Secretary Kathleen Sebelius stated “We’ve found there are certain people who will not pick up a newspaper. They won’t turn on the radio. They may not read the flyer that their doctor gives them. But they will check their text messages” [13].

**Fig. 1:**
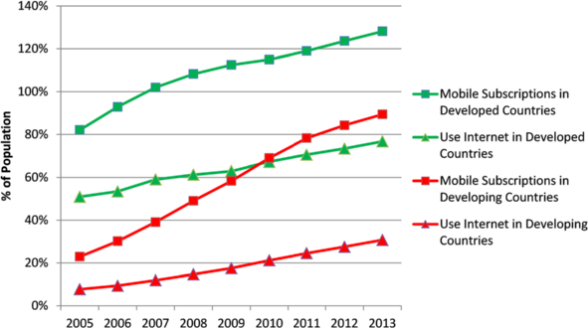
Reliance on cell phones increases with population vulnerability

In 2010, 59 % of adults in the US searched the Web for health information, most of which was then shared within the user’s social ecosystem [14]. Underserved populations affected by the digital divide have impeded access to the web and are more vulnerable to health disparities, but their use of text and picture messaging on cell phones is greater than that of more affluent demographics. An analysis of the Pew Health Tracking dataset for September 2012 confirms that individuals in the United States without Internet access are more likely to use text messaging than those with Internet access (Table 1) [15]. The disparity between Internet access and cell phone usage is also increasing in developing countries (Fig. 1) [16].

**Table 1:** Disparity of Internet access and text messaging by household income in 2012

Household income	<$10 K	$10 K–$20 K	$20 K–$30 K	$30 K–$40 K	$40 K–$50 K	$50 K–$75 K	$75 K–$100 K	$100 K–$150 K	>$150 K	Total
Text, Have Internet Access	8 %	8 %	10 %	11 %	9 %	18 %	14 %	12 %	10 %	100 % (*N* = 1507)
Text, Have No Internet Access	27 %	21 %	13 %	15 %	7 %	8 %	3 %	4 %	2 %	100 % (*N* = 123)

Text messaging is used by m-Health campaigns to deliver health preparedness information across the digital divide to vulnerable populations because it is supported by all cell phones including the simpler models (i.e., “feature phones” or “non-smart phones”) that are much less expensive to acquire and operate than smart phones [17], and which represent 84 % of the installed subscriber base and 66 % of current sales [18]. SMS does not require Internet access, and is typically the most resilient service in disaster scenarios. Because messages are pushed to the recipient, outreach is not contingent on the recipient’s initiative to search for and download content, and content forwarding via person-to-person messaging has been shown to promote a wider and faster dissemination of health information [19]. A drawback of SMS is that it only supports brief unformatted text (up to 160 characters), which severely constrains the information that a text message can convey.

Multimedia Messaging Service (MMS, i.e., picture and video messaging) is another resilient and popular cell phone data protocol that does not require Internet access or the installation of an “app”, with the main advantage over SMS that it conveys more data in a potentially more informative and easily assimilated format than just text. The MMS protocol pushes slideshows with audio narration and videos onto a cell phone, which can be played at any time even when there is no signal coverage, and are easily forwarded to friends and family. Through the use of illustrations and voiceover, a well-constructed MMS can convey information to individuals with reading disabilities more clearly and with greater retention than text alone [9].

Over the last 3 years, the cost of sending or receiving a MMS message has decreased from five times that of an SMS to roughly the same cost, and many carriers now bundle large or unlimited SMS/MMS quotas with voice plans. Among non-voice mobile usage, MMS is second only to SMS and is the fastest growing, with 249 billion MMS sent in 2010 representing an annual growth in traffic of over 47 % compared to 6.9 trillion SMS in 2010 representing 16 % annual growth [20].

In spite of the international ratification of the MMS protocol in 2001 and the growing multimedia capabilities of cell phones, MMS is rarely used in m-Health campaigns in Europe and the Americas. Here, the most common use of MMS is by individual subscribers sending a photo taken with their cell phone to another subscriber, whereas in China the most common use of MMS is by institutions broadcasting content to a broad audience [1].

**Fig. 2:**
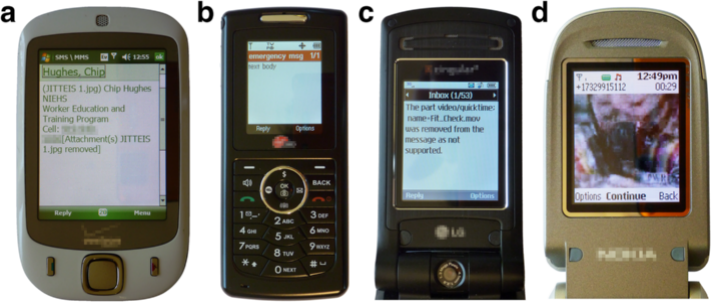
Commonly encountered MMS interoperability errors. **a** Carrier deleted an image from an incoming MMS. **b** Media received but phone cannot render it. **c** Carrier deleted a video from an incoming MMS. **d** Video re-sampled by carrier, plays poorly on phone

Market analysts agree that a major reason why United States institutions do not use MMS while they do use SMS (e.g., reminders and alerts) is because the implementations of the MMS protocol by device manufacturers and carriers lack the interoperability of their SMS implementations [8, 21]. Barriers to MMS interoperability stem from aggressive product differentiation by carriers and device manufacturers, and poor compliance with ratified protocols [22]. Collectively, modern cell phones support over a dozen mobile multimedia file formats and transmission protocols, but on average only 20 % of these will work with any given phone, and cell phones with non-overlapping multimedia capabilities are often encountered in an audience [23]. Consequently, lack of interoperability (Fig. 2) impedes the use of MMS in application-to-person (A2P) messaging campaigns, including m-Health outreach, because the sending institution has no assurance that messages will be received by a target audience that spans multiple carriers [24, 25].

MMS interoperability is greater in countries exhibiting less competition between carriers, such as where the dominant wireless carrier is not (or was only recently) privatized. Consequently, MMS-based campaigns have flourished in these countries. In China, for example, 70 % of MMS traffic in 2010 was A2P services including news and entertainment videos [26], and revenue from MMS exceeded that from SMS – the opposite of the situation in the US.

## Methods

### Summary

During the winter of 2011, CDC/OPHPR conducted a nationwide m-Health outreach campaign to evaluate the reach, ease of use, and appeal of short videos on preparing for severe winter weather that are pushed over-the-air to participants’ cell phones. The topic of winter weather preparedness was selected over other OPHPR outreach topics, including hurricane or tornado preparedness, because it is one of the most visited topics in the CDC public outreach web site, and hence the m-Health campaign might attract broader public participation. Outreach material from the winter weather preparedness web site was converted into nine mobile-ready videos.

The telecommunications company Cell Podium, located in the business incubator of the New Jersey Institute of Technology (NJIT), provided a middleware platform used by the campaign to circumvent the interoperability impediments to multimedia messaging. To achieve interoperability with older model cell phones, the middleware limited the duration of each video to no more than one minute.

OPHPR promoted the campaign on its winter weather preparedness web site and to subscribers on its govdelivery.com notification platform. The middleware includes an automated opt-in/out mechanism to comply with anti-spam legislation that prohibits sending messages to cell phones without the owners’ permission. Before receiving any outreach videos, each participant had to opt-in by either calling an automated voice recognition system or sending an email. During enrollment, each participant selected her/his preferred method for receiving videos: via cell phone messaging or via email.

The campaign broadcast nine videos following a bi-weekly schedule. If a participant enrolled in the campaign after the schedule had commenced, s/he would receive all prior videos automatically, thus ensuring each participant received all nine videos. The middleware logged all multimedia, text, and voice messaging activity to/from the middleware during enrollment and broadcasts for subsequent analysis. We used participants’ enrollment and delivery options to distinguish between participants with Internet access and those without Internet access (or preferring not to use the Internet).

### Ethics and approvals

**Fig. 3:**
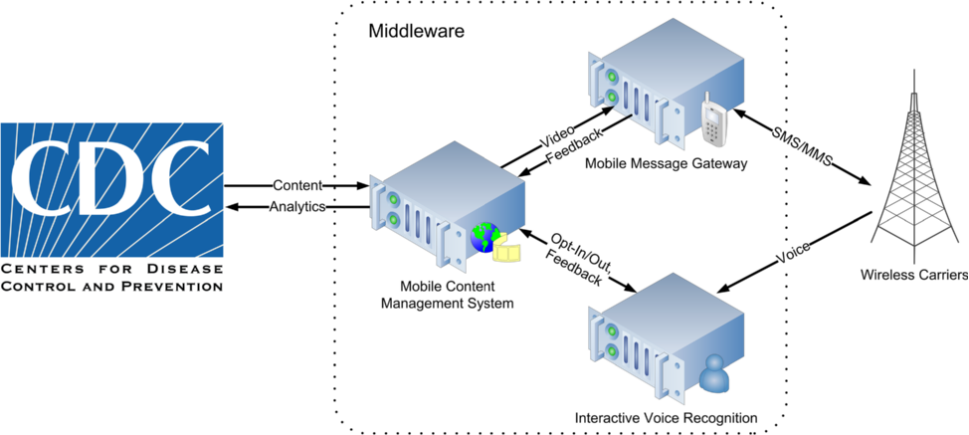
m-Health multimedia messaging middleware

The project was approved by CDC’s human subject review committee. No identifying information was collected from participants other than their cell phone number or email address, which were stored encrypted in a secure message gateway (Fig. 3) so the middleware would know to what number or address to send the media. OPHPR maintains a web-based public outreach campaign on the topic of severe winter weather public preparedness (http://emergency.cdc.gov/disasters/winter/), which served as the main source of original media assets for the m-Health campaign. Interns at NJIT and Cell Podium converted these assets into CDC-branded mobile-ready media, which CDC approved prior to campaign promotion and broadcast.

### Multimedia interoperability via middleware

We addressed the lack of mobile multimedia interoperability by deploying a middleware between CDC and the wireless carriers (Fig. 3). The middleware:

1. hosts CDC content in mobile-friendly formats (3GP, SMIL, and MP4, discussed in the next section),
2. pushes content to the cell phone of each participant through the participant’s carrier using a format (3GP, SMIL, or MP4) and protocol (MMS, SMS with link, or email) supported by the participant’s phone,
3. collects feedback received from participants via SMS, MMS, email, or voice,
4. collects feedback from wireless carriers when a message is undeliverable,
5. manages participant enrollment (opt-in and opt-out requests), and
6. maintains messaging activity log files from which it generates campaign analytics.

A content management system (a 2U rack mounted server) stored the multimedia and hosted the web server through which the campaign was managed. A gateway (a 4U rack mounted server) served as the interface with wireless carriers. Both the content management system and the gateway were hosted at a dedicated secure server farm, whereas the interactive voice recognition system was hosted on the cloud.

Middleware offers several significant advantages over cell phone “apps”: (1) users need not install any software, change any settings on their cell phones, or change devices, carriers, or service plans, (2) users of older “non-smart” phones are supported (apps can only be installed on smart phones), (3) no new software to learn or hardware to carry: recipients view courses using the same messaging and media player that came factory-installed in their phone, and (4) support for emerging mobile formats and protocols can be added easily in the middleware without involving the user or the carrier.

### Content development and scheduling

The posted CDC guidelines for winter preparedness [27] were converted into mobile videos. The three conversion steps and subsequent scheduling are described below.

#### Identify instructional design constraints

All carriers set a maximum on the size of a MMS message they will convey to a cell phone on their network. A carrier is not obligated to convey a MMS message that enters its network exceeding this size, and many carriers simply reject or drop such messages. The lowest cap among carriers is currently set at 300 K bytes per multimedia message, although some carriers with better infrastructure have higher limits. For example, in 2014 Verizon’s intra-carrier MMS cap was 500 K bytes, and is currently 1 M bytes.

MMS supports video in two formats. The ratified MMS protocol mandates that video be encoded using the 3GP codec at Quarter Common Intermediate Format (QCIF, 176 horizontal pixels × 144 vertical pixels) [28]. All cell phones with cameras have factory-installed the ability to receive via MMS and play such videos. The protocol also supports Graphics Interchange Format (GIF) animations and Adaptive Multi-Rate narrowband (AMR-NB) audio played back concurrently using the Synchronized Multimedia Integration Language (SMIL), all of which are supported by older pre-camera cell phones [29]. In the strict sense, this second format is an animation and not a video, but it can convey video content and modern web sites use it to display video thumbnails.

Three hundred kilobytes permit roughly 60 s of 3GP video with perceptually good audiovisual quality (40 kilobits per second). We thus set the constraints of each outreach message as one minute maximum duration, and all visual features had to be clearly discernible at QCIF resolution.

#### Storyboard and approval

**Fig. 4:**
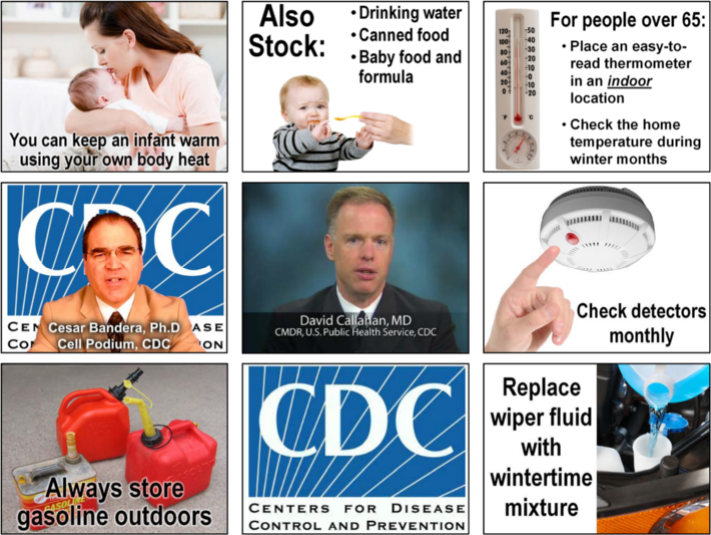
Screen shots of winter preparedness cell phone videos (all have voice narration)

Conforming to the above constraints, we created the storyboards for eight 1-minute videos on severe winter weather preparedness (Fig. 4, Table 2). The first seven storyboards were derived from CDC’s online winter preparedness guidelines, and demonstrated the adoption of existing image content for delivery mobile. The eighth video “Avoiding Carbon Monoxide Poisoning” was derived from a CDC public service announcement video, and demonstrated the adoption of existing video content for mobile delivery.

**Table 2:** Campaign video lineup

Video title:	Derived from:	Broadcast time (EST)
Preparing Your Home	http://emergency.cdc.gov/disasters/winter/beforestorm/preparehome.asp	2/4/2011 6:01 pm
Preparing Your Car	http://emergency.cdc.gov/disasters/winter/beforestorm/preparecar.asp	2/21/2011 3:00 pm
Supplies For Your Home	http://emergency.cdc.gov/disasters/winter/beforestorm/supplylists.asp	2/23/2011 1:19 pm
Supplies For Your Car	http://emergency.cdc.gov/disasters/winter/beforestorm/supplylists.asp	2/25/2011 11:55 pm
Heating Your Home Safely	http://emergency.cdc.gov/disasters/winter/duringstorm/indoorsafety.asp	2/28/2011 2:03 pm
Monitor Infant Body Temperature	http://emergency.cdc.gov/disasters/winter/duringstorm/indoorsafety.asp	3/2/2011 1:12 pm
Outdoor Safety	http://emergency.cdc.gov/disasters/winter/duringstorm/outdoorsafety.asp	3/4/2011 5:24 pm
Avoiding Carbon Monoxide Poisoning	http://www.cdc.gov/co/default.htm	3/7/2011 4:34 pm
Thank you for watching	N/A	3/9/2011 12:22 pm

OPHPR subject matter experts verified that the storyboards correctly summarized their winter preparedness guidelines. A ninth storyboard titled “Thank You for Watching” was also prepared, in which subscribers were asked to reply with comments on the campaign. PowerPoint was used to build the storyboards and to arrange assets in space and time including images, graphics, embedded videos, voice narration, sound effects, animations, and transitions.

#### Master video creation and conversion into mobile formats

Cell Podium staff converted each storyboard into a high-definition master video by exporting from PowerPoint. Post-production correction to audio, including noise reduction and volume normalization, was performed with Adobe Audition. Each master video was down-converted into a 3GP/QCIF file under 500Kbytes for feature phones with cameras, and a SMIL package with GIF and AMR-NB for older cell phones, using Adobe After Effects. Online copies of these videos can be viewed on http://www.youtube.com/user/cellpodium.

Smartphones come with more advanced codecs and web browser protocols that permit larger videos, and have larger screens with which to display video at higher resolution. To exploit this functionality of smartphones, each master video was also down-converted into an mp4 file at QVGA resolution (320 pixels horizontally by 240 pixels vertically) with a size of roughly 1Mbytes. During the m-Health campaign, the middleware pushed the 3GP versions to feature phones and the mp4 versions to Internet-enabled devices including smartphones and computers.

#### Schedule programming

The middleware was programmed with a broadcast schedule in which a video was sent to all participants enrolled in the campaign roughly every half week. To avoid annoying any participants, broadcast times were in the early afternoon and the schedule did not repeat the broadcast of any video. To ensure all participants received all nine videos, the middleware was also programmed to immediately push to any person enrolling in the campaign any videos that were previously broadcast. The first video of the campaign, “Preparing Your Home,” was made available to participants before the campaign was promoted to the public, and thus was delivered to a participant immediately upon her/his enrollment.

### Promotion and enrollment

In their effort to ban unsolicited mobile messages, the Telephone Consumer Protection Act of 1991 (47 USC § 227), the Controlling the Assault of Non-Solicited Pornography and Marketing (CAN-SPAM) Act of 2003 (15 USC § 7701), wireless carriers’ protection of their subscribers [30], and the best practices of A2P mobile services impose unique requirements and benefits to outreach via mobile messaging [31]. Participants must explicitly opt-in, and the procedure for opting-out must be simple and clearly communicated. If the campaign targets company-issued mobile devices, then the company must opt-in for all of its issued devices to participate. Lastly (and not unique to this study), a campaign that targets demographics with limited access to the Internet should use campaign promotion and enrollment techniques that are accessible to such demographics.

**Fig. 5:**
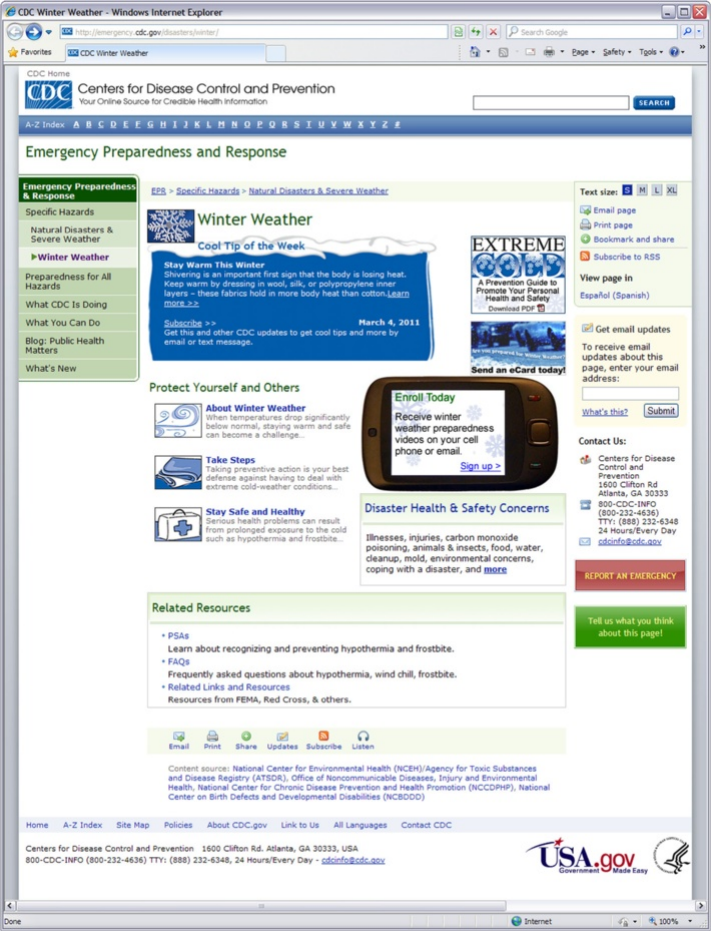
CDC guidelines for winter preparedness with invitation to mobile campaign

**Fig. 6:**
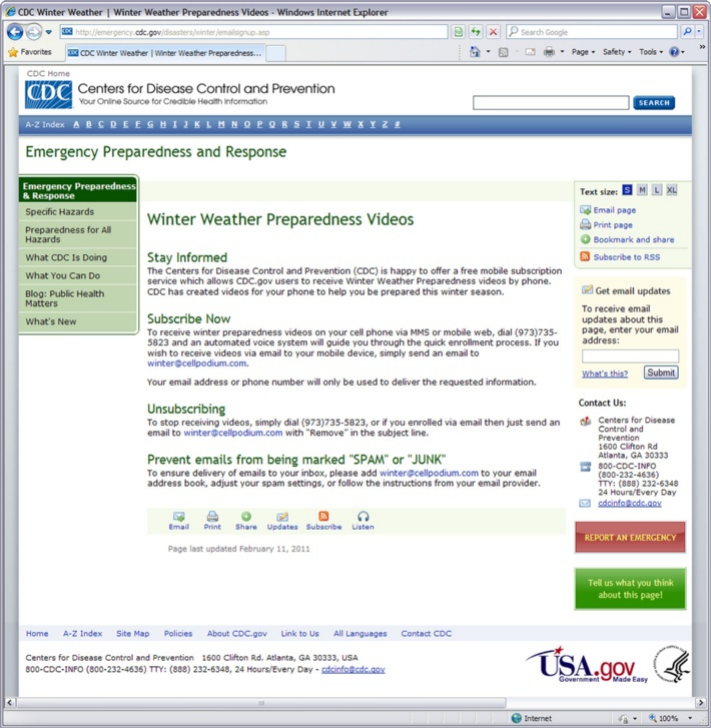
Web page describing the process for subscribing/unsubscribing from campaign

In this study, campaign promotion also had to comply with CDC outreach protocol which required that campaign promotion be CDC-branded and approved by the OPHPR Communications Branch. OPHPR approved the use of its web page on winter preparedness as a platform for campaign promotion and, if necessary, its GovDelivery.com email notification system. The latter is discussed in the section on Formative Assessment. The CDC web page on winter preparedness was updated with an invitation to participate in the mobile multimedia outreach campaign (Fig. 5) and a link to an additional web page (Fig. 6) containing details on the campaign, its schedule, and procedures to opt in and out. Participants unfamiliar with messaging or without Internet-enabled phones were invited to enroll in the campaign by making a voice call from their cell phone to a dedicated phone number.

**Fig. 7:**
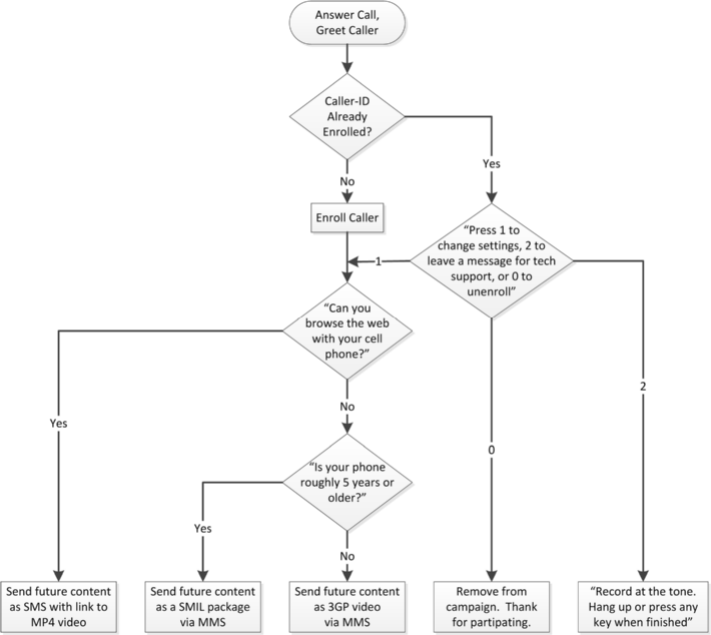
Interactive voice recognition program

Callers were greeted by an interactive voice recognition system programmed to retrieve the caller ID, compare it with the list of previous callers who had enrolled, and ask any first-time caller if s/he is able to surf the web with the cell phone or if the phone is very old (Fig. 7). During broadcasts, the middleware pushes videos to each cell phone using the format and delivery protocol best suited to the participant’s response to this question. Participants were also invited to enroll in the campaign via email; during subsequent outreach broadcasts, the middleware pushes videos to these email addresses as links to the MP4 files.

### Analytics collection

Throughout the campaign, we monitored communications into the middleware from carriers in order to detect if any messages were being rejected by any carrier or handset. We also monitored system logs, which included the exact times of broadcasts to each participant, and the opt-in/out interaction.

Campaign participants with Internet access receive messages (SMS or email, depending on how they enrolled) with links to high-quality MP4 versions of the campaign videos. When the participant clicks on the link, client software (typically a web browser) submits to the middleware an HTTP request for the MP4 file, downloads the file onto the participant’s device, and plays the file. As part of the HTTP request to the server, the client software identifies itself and the device’s operating system, but not the phone number of the participant. Starting in the morning of March 7, 2011, Cell Podium began recording this detailed Internet traffic between the middleware and the participant’s client software, and was able to classify the software accessing the “Avoiding Carbon Monoxide” and “Thank You for Watching” videos.

### Formative assessment

**Fig. 8:**
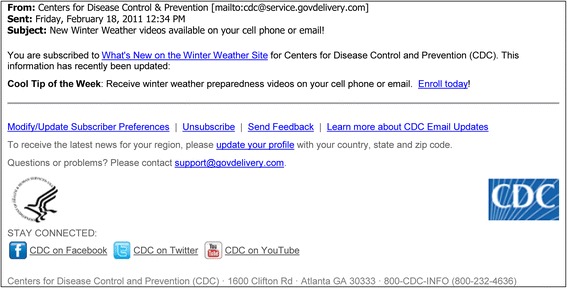
GovDelivery.com email announcement of cell phone video campaign

On February 16, 2010, OPHPR staff posted the campaign promotion and enrollment information on the CDC web site (Figs. 5 and 6). No enrollment activity occurred during the following 2 days, indicating a need for pushed-based campaign promotion as opposed to the traditional pull-based web promotion. Moreover, campaign promotion via a web page was not expected to enroll many users with limited Internet access. CDC maintains an email list of individuals (at that time roughly 30,000) who requested via GovDelivery.com to be notified of updates to the winter weather preparedness web site. At 12:30 pm EST on February 18, OPHPR sent an invitation email via GovDelivery.com to these individuals (Fig. 8), resulting in immediate enrollment activity.

## Results

**Fig. 9:**
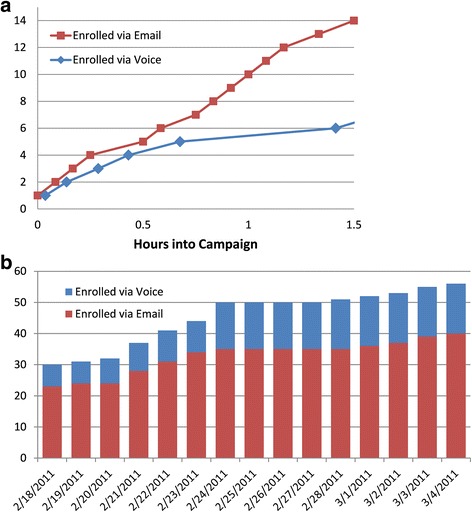
Enrollment activity during the first ninety minutes (**a**) and fifteen weeks (**b**) of the campaign

A total of 504 videos were delivered by the campaign. Fifty-six people participated in the 5-week campaign, of which 20 enrolled within 90 min of the GovDelivery.com email (Fig. 9). This enrollment activity demonstrates the importance of “push-based” content delivery for both outreach and campaign promotion. Forty participants enrolled via email on computers and Internet-enabled cell phones, and 16 enrolled on non-Internet enabled cell phones. Only two people un-enrolled prior to the completion of the campaign, both non-mobile users, and no error messages were received from carriers or mobile phones. No participant indicated they had an older phone, so all the videos pushed by the campaign were either in MP4 or 3GP format, and no videos were sent in SMIL format.

In March, the ability of the middleware to analyze message traffic details was completed and put into operation, permitting a more refined classification of the devices to which videos were sent. Between 3/17 and 3/19, inclusive, the “Avoiding Carbon Monoxide” and “Thank You for Watching” videos were served 108 times. Message traffic analysis revealed that 15 of these videos were served not to participants, but to web crawlers (“Bot” in Table 3). A bot is a program that automatically surfs the web, hopping from link to link and collecting data on every page it visits. Search engine companies typically use bots to collect information about active web sites and to maintain their search results current. Among these bots were web crawlers from Yahoo, MSN, Google and Twitter.

**Table 3:**
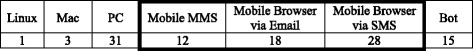
Videos sent between 3/17 and 3/19 classified by participant platform

Neither CDC, NJIT, nor Cell Podium posted links to the videos on their web sites. We suspect the bots found links to the videos in the history of the web browsers of participants that enrolled in the campaign via the Internet (e.g., via participants using a web browser to access campaign emails).

Excluding video retrievals by these bots, the campaign served 93 videos to enrolled participants between 3/17 and 3/19, inclusive. Of these 93 videos, 53 were served to participants who enrolled via email. Of these 53 emailed videos, one third was served to mobile devices and the rest to desktop/laptop computers (“Mobile Browser via Email” versus “Linux”, “Mac”, and “PC” categories in Table 3). Overall, most (58 out of 93) videos were served to mobile devices (highlighted in Table 3). Note that the videos in the “Mobile MMS” category (21 % of cell phone users) were not retrieved by participants with Internet-enabled devices, but instead were pushed onto mobile devices using the MMS protocol that does not require Internet connectivity.

**Fig. 10:**
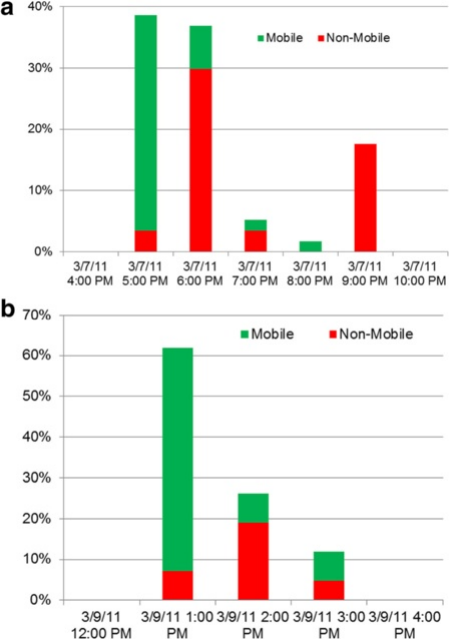
Mobile device users watched the "Carbon Monoxide" (**a**) and "Thank You for Watching" (**b**) videos sooner than non-mobile users

Internet traffic analysis also revealed the elapsed time from when participants received a message with a new video (3/7/2011 4:30 pm EST for “Avoiding Carbon Monoxide Poisoning” and 3/9/2011 12:20 pm EST for “Thank You for Watching”), and when they actually viewed the video (Fig. 10). On average, users of mobile devices viewed videos 82 min after receiving them, whereas users of desktop computers viewed videos 173 min after receiving them.

Of the 56 participants who enrolled in the campaign, only two un-enrolled before the end of the campaign. To finalize the campaign, a “Thank You for Watching” video was broadcast on March 9. This 30 s video asked subscribers to submit feedback by simply responding to the video itself, i.e., via text message, multimedia message or email. Two responses were received. One was a simple SMS saying, “provide more videos.” The other response was longer:

*My company* [deleted for anonymity] *works with individuals who have developmental disabilities. I found the courses on winter preparedness - “Outdoor Safety”, “Supplies for Your Car”, and “Supplies for Your Home” to be beneficial both for training staff and for educating the individuals we serve. I am constantly looking for interesting mediums through which I can present pertinent information in an interesting way. Cell Podium courses help me in this endeavor.*

*I would be interested in courses on food safety, home safety, infectious diseases, diabetes, infection control/blood borne pathogens, healthy living -- subjects like exercise, weight loss, what to do to prevent health issues.*

*I appreciate what you are doing and look forward to more Cell Podium courses.”*

## Discussion

The main lessons learned from this study pertain to the underlying MMS technology, m-Health campaign promotion, and the user experience. The following three sections address each of these aspects. A fourth section “Practical Implications” discusses how the results of this project are being used in subsequent CDC m-Health campaigns.

### MMS protocol

The campaign demonstrated to CDC that it could push multimedia archived on its web site, including illustrations and public service announcements, to people without Internet access. All multimedia broadcasts were received successfully because no carrier reported any message as undeliverable; this is particularly noteworthy because no limits were placed on who could enroll or with what mobile device. The constraints imposed by the MMS protocol on the duration and resolution of content (under 60 s at QCIF) were sufficient to convey each topic in a single video message.

Another constraint imposed by the MMS protocol is that the only analytics automatically collected from a recipient’s cell phone is the successful delivery of the MMS. The middleware does not automatically detect if a participant forwards the MMS to another person, because this is a peer-to-peer operation involving their carriers and not the middleware; that information could be solicited by explicitly asking that question in the video itself, and responses would be optional.

### Campaign promotion

Having solved the technical obstacles in mobile multimedia interoperability, this project was impacted by the more traditional impediment to public health outreach, namely the effectiveness of campaign promotion. Of the two campaign promotion mechanisms approved for the study by OPHPR, the most successful was the February 18^th^ email from GovDelivery.com to its list of subscribers who had requested to receive notifications of changes to the CDC winter weather preparedness web site. The other mechanism, namely the change to the CDC web site two days earlier, yielded no enrollment prior to the GovDelivery.com email. Web crawlers also support campaign promotion to individuals with Internet connectivity by displaying campaign content in search results. However, GovDelivery.com subscribers and viewers of CDC and search engine websites have Internet access and are not necessarily representative of vulnerable demographics.

This study highlights the need for techniques with which to promote MMS campaigns targeting demographics without Internet. One technique is person-to-person messaging, which has been used successfully to promote awareness of SMS-based m-Health campaigns [32]. Future CDC multimedia m-Health campaigns will include videos encouraging participants to forward enrollment instructions (included in the video) to contacts. The ability to include this large amount of information in one forwarded message is a benefit of MMS over SMS.

Some countries permit the broadcast of unsolicited SMS messages inviting the recipient to opt into a mobile campaign. This approach reduces the enrollment effort to a simple click (a reply to the invitation) that triggers an instant response from the system and possibly an incentive (e.g., a retail coupon code). However, this approach is considered spamming in the United States.

Simple signs and flyers can promote a MMS campaign and a simple SMS-based enrollment process (i.e., text a keyword to a dedicated number); while this approach does not have the scalability of a mass broadcast, it still benefits from the simplicity and instant gratification of the enrollment process. Future CDC multimedia m-Health campaigns using the middleware will include enrollment via SMS.

Another enrollment mechanism is via quick response (QR) code. Commonly used to direct a cell phone’s web browser to a URL encoded in the 2-D visual pattern, a QR code can also invoke a cell phone to send a text message to a number, both which are encoded in the visual pattern. This approach does not require Internet access, but does require an advanced phone with a QR code reader.

### User experience

Regulatory requirements reduce the amount of spam via mobile messaging and increase the likelihood that SMS and MMS will not be ignored. Whereas roughly 80 % of 2011 email traffic was spam [33], “only” 4.5 billion of the eight trillion text messages received in 2011 (0.06 %) were considered spam [34]. The open rate of text messages is 90 % within 15 min of receipt, whereas for email it is only 20–25 % within 24 h of receipt [35].

Content pushed to cell phones is intrusive in the sense that it captures attention, but is well received so long as it is relevant to the user. Campaign promotion must set expectations clearly so that participants are not disappointed or annoyed by the campaign [36]. In this respect, promotion may be more important to mobile multimedia public health outreach campaign enrollment than to other types of public health outreach [37].

Given a greater intolerance to unwanted mobile messages when compared with email spam, this study took efforts - beyond meeting the regulatory requirements for A2P mobile messaging - to avoid its broadcasts being a perceived as nuisance. First, the time between broadcasts was set to the maximum permitted by the fact that all videos had to be sent before the end of the winter weather season. Consequently, on average two videos were sent per week.

Second, communications from participants including replies to the mobile messages and voice calls was monitored, particularly for attempts to opt-out. Even though the opting-out procedure was designed to be simple, it is conceivable that a participant could have performed the procedure incorrectly, in which case the system would have logged the attempt but not recognize it as such. Sixty messages were received during the campaign: 56 enrollments, two un-enrollments, and the two positive comments.

Third, broadcast content focused on practical recommendations within the narrowly-defined theme of the campaign. Moreover, the 60-second constraint on high-quality MMS videos forced each campaign video to be brief. Prior studies show that m-Heath participants prefer practical advice with immediate relevance over general distance learning or prescriptive persuasion [23, 38]. Overall, the campaign’s high retention rate (54/56 = 96.4 %) and positive (albeit sparse) feedback suggests that the mobile videos were welcomed by participants. Moreover, the quicker viewing of media sent to mobile devices (versus to non-mobile devices, Fig. 10) suggests that the broadcasts were not simply ignored.

### Practical implications

In September 2012, the OPHPR Emergency Operations Center (EOC), CDC’s command center for monitoring and coordinating global emergency public health response activities, launched a two-year program that uses the middleware described in this paper to support two kinds of campaigns: mobile multimedia health and preparedness outreach to civilians that include vulnerable populations without Internet access, and communications with CDC personnel deployed in areas with compromised Internet infrastructure.

**Fig. 11:**
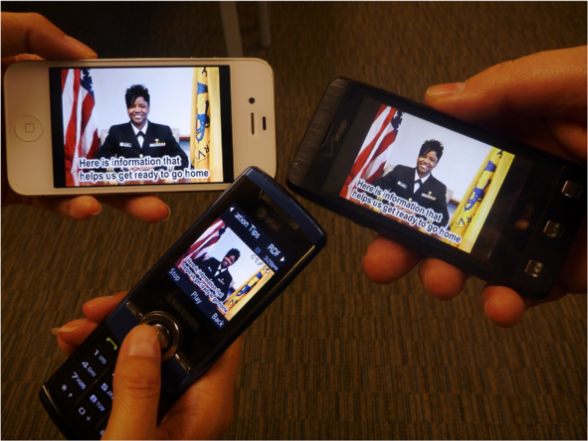
Different cell phones playing a RDF-3 video during Hurricane Sandy

An example of the latter occurred during the aftermath of Hurricane Sandy, when U.S. Public Health Service Rapid Deployment Force-3 (RDF-3) arrived in New Jersey to establish Federal Medical Stations for patients who needed medical services not provided at regular shelters [39]. Power outages interrupted most cell phone towers, hampering wireless broadband and even voice calls on cell phones. Moreover, wind damage took out conventional wired Internet. However, SMS and MMS, which require less power and bandwidth than wireless voice or broadband, were reliable. The EOC used the middleware to broadcast logistics videos with mobilization instructions to the cell phones of RDF-3 personnel (Fig. 11), and help coordinate response operations.

In addition to the m-Health functionality discussed in this paper, the EOC program is investigating (1) the ability to push multimedia to cell phones overseas, (2) automatic conversion of existing web-based multimedia into mobile broadcast, and (3) situation awareness. The latter involves exploiting the bi-directionality of multimedia messaging to collect imagery and videos sent by participants via MMS to the middleware.

### Limitations

A key limitation of the study is the method used to promote the campaign. GovDelivery.com subscribers and viewers of CDC and search engine websites have Internet access and are not necessarily representative of vulnerable demographics. GovDelivery.com subscribers include healthcare stakeholders such as training and community organizations. Another limitation is the small amount of information collected automatically from cell phones to which SMS or MMS are sent, which only consists of an indication by the participant’s phone company that a message could not sent (e.g., if a person tried to enroll on a land line). This limitation is due to the SMS and MMS protocols themselves, which are less sophisticated than the HTTP protocol.

## Conclusions

This work presents a campaign designed to broadcast multimedia to cell phones of different models and carriers without requiring them to have Internet connectivity. By using the MMS protocol and MMS players that are factory-installed in cell phones, subscribers did not have to change any settings on their cell phones to receive or view the content. To date, this is the only mechanism that will push multimedia to cell phones without Internet connectivity, as is commonly the case among vulnerable populations.

Mobile video can be as much a staple of m-Health campaigns as text messaging. Both mechanisms are supported by all cell phones regardless of Internet connectivity. Adapting existing content into video is often easier than complying with the severe content constraints of SMS, and low-cost or free video authoring tools are now commonplace. For example, recent versions of Microsoft PowerPoint will export to video, rendering all embedded animations, voice-over narrations, and “picture-in-picture” videos, making this ubiquitous tool a platform for storyboarding and production. Taking the appropriate technical precautions to circumvent the lack of handset interoperability, m-Health campaigns can exploit the larger information content and retention of multimedia [9].

## Abbreviations

3GP: multimedia container format for cell phones ratified by the third generation partnership project
AMR-NB: adaptive multi-rate narrow band format for voice-quality audio
CDC: centers for disease control and prevention
DHHS: Department of Health and Human Services
EOC: emergency operations center (CDC/OPHPR, Atlanta, GA)
GIF: graphics interchange format for images and image sequences
m-Health: healthcare delivered via the patient’s cell phone
MMS: multimedia message service
OPHPR: CDC office of public health preparedness and response
QCIF: quarter common intermediate format (176 horizontal pixels × 144 vertical pixels)
QVGA: quarter video graphics array (320 horizontal pixels × 240 vertical pixels)
RDF: U.S. Public Health Service Rapid Deployment Force
SBIR: small business innovation research
SMIL: synchronized multimedia integration language
SMS: short message service (mobile text messaging)
